# Suppression of abnormal grain growth in K_0.5_Na_0.5_NbO_3_: phase transitions and compatibility

**DOI:** 10.1038/s41598-019-56389-9

**Published:** 2019-12-24

**Authors:** Patricia Pop-Ghe, Norbert Stock, Eckhard Quandt

**Affiliations:** 1 Inorganic Functional Materials, Insitute for Materials Science, Faculty of Engineering, Kiel University, 24143 Kiel, Germany; 2 Inorganic Chemistry, Insitute for Inorganic Chemistry, Faculty of Mathematics and Natural Sciences, Kiel University, 24118 Kiel, Germany

**Keywords:** Energy science and technology, Materials science

## Abstract

This work presents the suppression of abnormal grain growth in bulk ceramic *K*_0.5_*Na*_0.5_*NbO*_3_ (KNN). The suppression is enabled by precise control of the starting powder morphology through match of milling and calcination duration. A comparative temperature-dependent analysis of the resulting sample morphology, phase transitions and related electronic material properties reveals that abnormal grain growth is indeed a major influence in material property deterioration, as has theoretically been suggested in other works. However, it is shown that this abnormal grain growth originates from the calcined powder and not from sintering and that all subsequent steps mirror the initial powder morphology. In specific, the results are discussed with respect to the predictions of the compatibility theory and microstructure. Despite the material’s multi-scale heterogeneity, the suppression of abnormal grain growth allows for the achievement of significantly improved functional properties and it is reported that this development is correctly predicted by the compatibility theory within the borders of microstructural integrity. It could be demonstrated that functional fatigue is strongly minimised, while thermal and electronic properties are improved when abnormal grain growth is suppressed by powder morphology control.

## Introduction

Lead-free ceramics are intensely investigated^1^, as the most widely used piezoelectric material lead zirconate titanate (PZT) and other high performance piezoelectrics like Pb(Mg_1/3_Nb_2/3_)O_3_ – PbTiO_3_ (PMN-PT)^2,3^ contain lead, which is highly toxic for humans and for our environment. This has been recognised by the EU by the Directive 2011/65/EU, which “prohibits the use of lead in electrical and electronic equipment placed on the market” by 2021^4^. Among those, there are two inorganic perovskite (ABO_3_) material systems which are regarded as most promising in regard to a substitute: the barium titanate (BTO) and the potassium sodium niobate (KNN) system. Those materials exhibit a broad range of effects and can be used in sensors and actuators, as well as in caloric applications aiming at environmentally friendly cooling technologies^5^. They do not only exhibit piezoelectricity, but also first-order phase transitions that can potentially be exploited in electrocaloric (EC) cooling^6^. However, comparably low achievable piezoelectric performance in BTO-based composites and low Curie temperatures in high performance calcium and zirconium doped BTO, as well as poor reproducibility in KNN have hindered their extensive application ever since the 1950s^7,8^. Between those two systems, KNN is less well understood due to its multi-scale heterogeneity^9,10^ and additionally shows poor sinterability^11,12^, which has been a major barrier to the application of the material. It was not until Saito *et al*.^13^ reported a very high piezoelectric coefficient of 416 *pC*/*N* in textured KNN ceramics in the year 2004 that research on KNN intensified again. On the basis of a strong effort of the scientific community to make this material usable in applications, KNN has by now been shown to exhibit very high Curie temperatures and excellent thermal stability of the piezoelectric performance both of which are decisive criteria for applications^14–16^. Still, even though the material is continuously improved^17–19^ the full potential of the material remains unexploited and subject of intense research^20^.

There is a broad variety of techniques which have been applied to improve the fabrication of bulk KNN in terms of reproducibility, as well as of material performance. Among those are a very high number of different processing techniques including unconventional approaches like microemulsion mediated synthesis^21^, hot isostatic pressing^22^, hydrothermal synthesis^23,24^ and spark plasma sintering^25^, as well as conventional solid state synthesis^26–28^. In terms of compositional chemistry doping approaches range from the simple change of alkali metal ratio to the addition of ternary and more complex alloys^29–31^. Still, bi- or even multimodal grain growth was reported for most of the synthesised materials^32–34^, although its suppression was seldom the centre of attention. Recently, even the coexistence of “KNbO_3_-like and NaNbO_3_-like grains” in calcined KNN powders was observed by Thong *et al*. and attributed to abnormal grain growth and composition heterogeneity^35^. Composition homogeneity is highly desirable to explore the material’s potential therefore several approaches have been developed to suppress heterogeneities by solid solution precursor methods^36^ or perovskite methods^37^. Still, abnormal grain growth or development of secondary phases persisted to occur^38–40^.

The lack of experimental data on potassium carbonate and other alkaline carbonates can partially be explained by their high hygroscopicity. Potassium carbonate (*K*_2_*CO*_3_) almost immediately transforms to sesquihydrate *K*_2_*CO*_3_·1.5 *H*_2_*O* at contact with air^41^ and the X-ray diffraction (XRD) peaks of this sesquihydrate are overlapped with XRD peaks of *γ*−*CO*_3_. Therefore, an easy reproducibility of the starting conditions is often hampered. Thus it is acknowledged that the fabrication of KNN requires enhanced safety precautions, either in form of glove box handling of the precursor powders *K*_2_*CO*_3_, *Na*_2_*CO*_3_ and *Nb*_2_*O*_5_, handling under gas atmosphere or heat treatments prior to processing to vaporise remains of water. A second consequence of this hygroscopicity is that the KNN fabrication process needs to be monitored right from the beginning to ensure reproducibility. Although it is known that the starting materials have a crucial effect on the resulting bulk morphology the influence of fluctuations in calcined KNN powder has often been analysed separately^21^ or has even been neglected, as the origin of insufficient material performance has been attributed to the multi-scale heterogeneity occurring *during* sintering. Among these multi-scale heterogeneities, the different diffusion velocities for the A-site elements potassium and sodium^11,42^ and the varying vapour pressures for potassium and sodium over KNN^11^ are the most prominent differences. But also differing KNN morphologies have been reported depending on the crystal structure of the niobium oxide, which is used^43^, as well as an increased chemical heterogeneity due to diffusion reactions of added ternary alloys^35^ and rising chemical complexity. At 1100 °C, a temperature well within the sintering regime, the diffusion coefficient of sodium in polycrystalline niobium is 51.2310^13^ *cm*^2^/*s* compared to a diffusion coefficient of 21.710^13^ *cm*^2^/*s* for potassium under the same conditions^42^. Considering that diffusion reactions are limited by the species with lower diffusion speed, consequently the KNN calcination is governed by the amount of potassium within the composition. At the same time, the higher diffusion rate in sodium is suggested to be one of the various reasons for abnormal grain growth, as “NaNbO_3_-like grains” might just grow faster than stoichiometric KNN grains^35^. It is worth noting that the diffusion velocities measured by Karpman *et al*. decrease strongly for the single crystal, which means that there is significant grain boundary diffusion for the small alkali metal atoms in niobium. Additionally, the vapour pressure of potassium has been demonstrated to be higher than that of sodium^44^, while it has to be taken into account that the sintering regime is very narrow in KNN, as it is bordered by the solidus line at around 1150 °C^11^. Moreover, even the sintering atmosphere has been suggested to influence grain morphology in KNN through coarsening of grains and the correlated change of edge free energy^45^. Notwithstanding that chemical heterogeneities are often tolerated and incorporated into the analyses in a descriptive way, their control is of special importance as alkali deficiency is a frequently reported problem in KNN processing. Taking all of this into account, the fabrication of high quality potassium sodium niobate remains a major challenge and demands for deeper knowledge and new design rules. As a consequence, this work combines KNN fabrication with the theoretical model of phase compatibility^46,47^, which has already been proven to be suitable for the prediction of ultra-low fatigue metal compositions with very narrow hystereses in metals^48,49^. Different aspects of the multi-scale heterogeneity will be discussed to attribute for their individual influences, while the theory of phase compatibility is applied to confirm promising material compositions. The compatibility theory aims at the improvement of fatigue characteristics of a material through a set of mathematical conditions, which can be satisfied by composition engineering. This set of rules is widely applied in metal compositions in high-cycle medical applications e.g. nickel titanium based composites^50,51^.

## Results and Discussion

### Preparation of the powder

**Figure 1:**
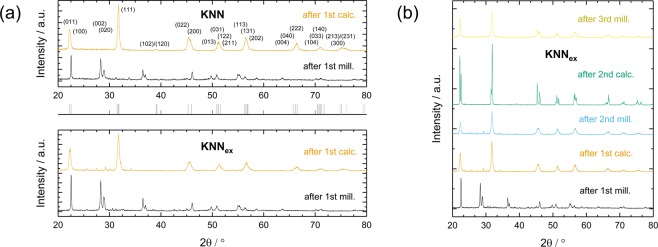
Processing of KNN and KNN_ex_: powder batches and resulting diffractograms for KNN_ex_ after each single processing step. (**a**) Comparison of KNN (indexed pattern^60^) and KNN_ex_ powder batches after the first milling (after 1^st^ mill.) and the first calcination (after 1^st^ calc.) with space group *Amm*2 indexing below (grey lines). (**b**) X-ray diffraction was performed on the KNN_ex_ powder batch after every processing step and the powder sample was examined for secondary phases with respect to phase purity.

In the following, KNN and KNN_ex_ will denote *K*_0.5_*Na*_0.5_*NbO*_3_ samples without addition of excess alkali metals and with addition of alkali metals respectively. In particular, KNN_ex_ will describe samples with an addition of 5 mole-% excess potassium and 15 mole-% excess sodium throughout this work as this specific amount of (A-site) excess alkali metal addition has shown the most promising improvement^52,53^ (cf. supplementary information). This can partially be explained by the slower reaction rate and the correlated restrictive role potassium plays in the calcination of KNN. For the other part, it seems contradictory as potassium has the higher vapour pressure. However, in the event of other tested additional amounts of alkali metals samples demonstrated deteriorated material properties^52^ (cf. supplementary information). A B-site excess (niobium excess) is not beneficial in regard to compositional homogenisation, which has been demonstrated by other works^54–56^, hence this work concentrates on A-site excess. It follows that the influence of diffusion on the final chemical composition is more significant than the contribution by evaporating material. Also, it is very probable that there are regions of accumulated sodium in or on the sample. After the first ball milling and calcination of KNN and KNN_ex_ respectively the XRD analysis shows additional XRD peaks between 23° and 29° to different extents depending on the composition of the sample (Fig. 1(a)), which correspond to secondary and multi niobate phases. The diffractograms were obtained with a Rigaku SmartLab 9 kV X-ray diffractometer with conventional CuK_α_ radiation. Undesirable multi niobate phases may generally occur between the (001)/(011) reflections at 22.5° and the (020)/(002)/(111) reflections at 31.8°, as well as between the (020)/(002)/(111) reflections and the (102)/(120) reflections at 39° based on *Amm*2 space group indexing at room temperature (cf. Fig. 1(a)). Especially a reflection at 28.1° is of importance as it originates from a moisture sensitive phase. To account for the necessary safety precautions and the material’s hygroscopicity mentioned above all of the precursor powders were dehydrated prior to usage. It is noted that the introduction of excess alkali metals causes an intensified occurrence of secondary phases after the first calcination. By choosing the right combination of ball milling duration, calcination and repetition times the development of those multi niobate and secondary phases can be suppressed (Fig. 1(b)). In detail, this is given for two repetitions of milling and calcination and an additional final (third) milling to ensure grain size homogeneity in the powder. If another calcination is applied undesired secondary phases occur again (cf. supplementary material), less repetitions are not sufficient to control the development of secondary phases either. This holds true for all tested undoped KNN compositions under the given conditions. We attribute this to the importance of the planetary milling, which ensures the size reduction of bigger particles and overall homogenisation of particle sizes, as has been found by other works^35^. However, every time the precursor powder is subjected to a calcination the mentioned inequalities between the different A-site elements are triggered due to the elevated temperature, thus it is unfortunate to end the process flow with a calcination. In the end of the powder fabrication process, the material is most sensitive towards changes due to the fragile KNN equilibrium state, therefore the appearance of secondary phases after the third calcination is interpreted as transgression of the KNN equilibrium.

### Abnormal grain growth

**Figure 2:**
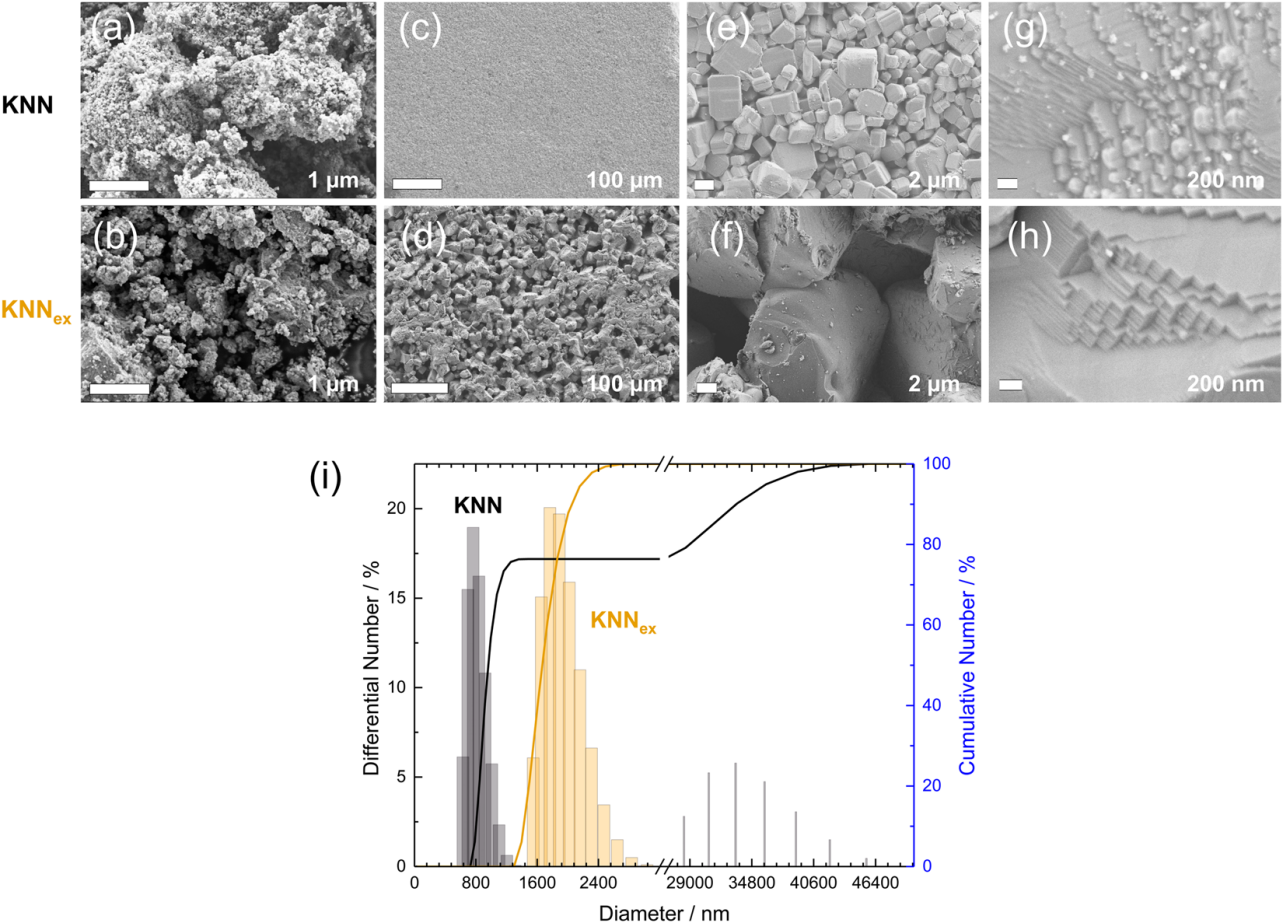
Origin of abnormal grain growth. (**a–h**) Scanning electron microscopy analysis of the powder batches from Fig. 1(a,b) and resulting sintered bulk ceramics at different magnifications. (**a,b**) Powder batches right before sintering. (**c–h**) Resulting KNN and KNN_ex_ bulk ceramics respectively. (**i**) Dynamic light scattering (DLS) measurement of KNN and KNN_ex_ powder batches right before sintering. The KNN powder shows a bimodal grain size distribution, while KNN_ex_ exhibits a monomodal distribution.

As has been described by Thong *et al*. and other groups^35,39^ abnormal grain growth is assumed to be one of the main obstacles in the reliable fabrication of high performance KNN. It was shown that even repeated ball milling and calcining of the samples, as well as different processing techniques^12^ could not suppress abnormal grain growth^35^. The reason for this abnormal grain growth is usually thought to lie within the different diffusion velocities and vapour pressures of potassium and sodium during the sintering process. Figure 2(a–h) show the SEM examination of KNN and KNN_ex_ powders and bulk ceramics prior to and after sintering, wherein Fig. 2(a,b) show the powder morphologies prior to sintering and all of the other images show the bulk pellet results after sintering in air. In detail, the powder comparison in Fig. 2(a,b) reveals agglomerates in both cases. The KNN powder is finer in contrast to the very coarse KNN_ex_ powder, but on the other hand the KNN_ex_ powder seems more homogeneous. This impression is substantiated when the sintering results in Fig. 2(c–h) are considered. Indeed, the larger and more homogeneous powder particles result in a homogeneous microstructure consisting of comparably large, loosely connected grains (Fig. 2(d,f)), while the fine powder leads to denser results which show the typical abnormal grain growth in KNN (Fig. 2(c,e)) with smaller and bigger grain sizes occurring randomly. However, major differences in the powder could not be verified reliably by the SEM examination due to the agglomeration of particles and restricted possibilities for adequate preparation. Therefore, the origin of bimodal grain growth could not be pointed out. As a consequence, dynamic light scattering (DLS) measurements were performed on the powder batches after the third milling (before sintering), which are shown in Fig. 2(i). A bimodal grain distribution resulting from abnormal grain growth is clearly demonstrated by this measurement, which can be recognised by the existence of two diameter distributions occurring in the KNN and the absence of it in the KNN_ex_ precursor powder respectively. This proves that abnormal grain growth originates from the calcined precursor powder in the presented case. In particular, the obtained grain sizes for excess alkali addition are significantly higher than for the KNN powder with a mean diameter of *d*_KNNex_ = 2.06 μm for KNN_ex_ and *d*_KNN_ = 1.1 μm for KNN powder respectively. This is in general agreement with other works on the particle size of calcined KNN powders^21^. What is more is that even on the nanoscale the differences are extensive. A change in preferred orientation can indeed be proven by microscopy (cf. Fig. 2(g,h)). In the KNN_ex_ sample a clear layer-by-layer step formation can be observed, which is not present in the KNN sample. These structures form due to the combined growth of singular (100) and non-singular (110) faces as has been predicted in crystalline growth at surfaces by Sangwal and co-workers^57^. In detail, the very low density of the samples, which can be presumed from Fig. 2(d) is undesirable, but in general agreement with diverse studies on the application of different sintering atmospheres^58,59^. Recently, it has been shown that sintering in air causes the least weight loss (alkali evaporation) in comparison to other tested atmospheres, but also reaches rather low densities^58^. This is attributed to the change in edge free energy, which is initiated by coarsening of grains^45^. These findings are in very good agreement with the results presented in this work.

### Excess alkali KNN

**Figure 3:**
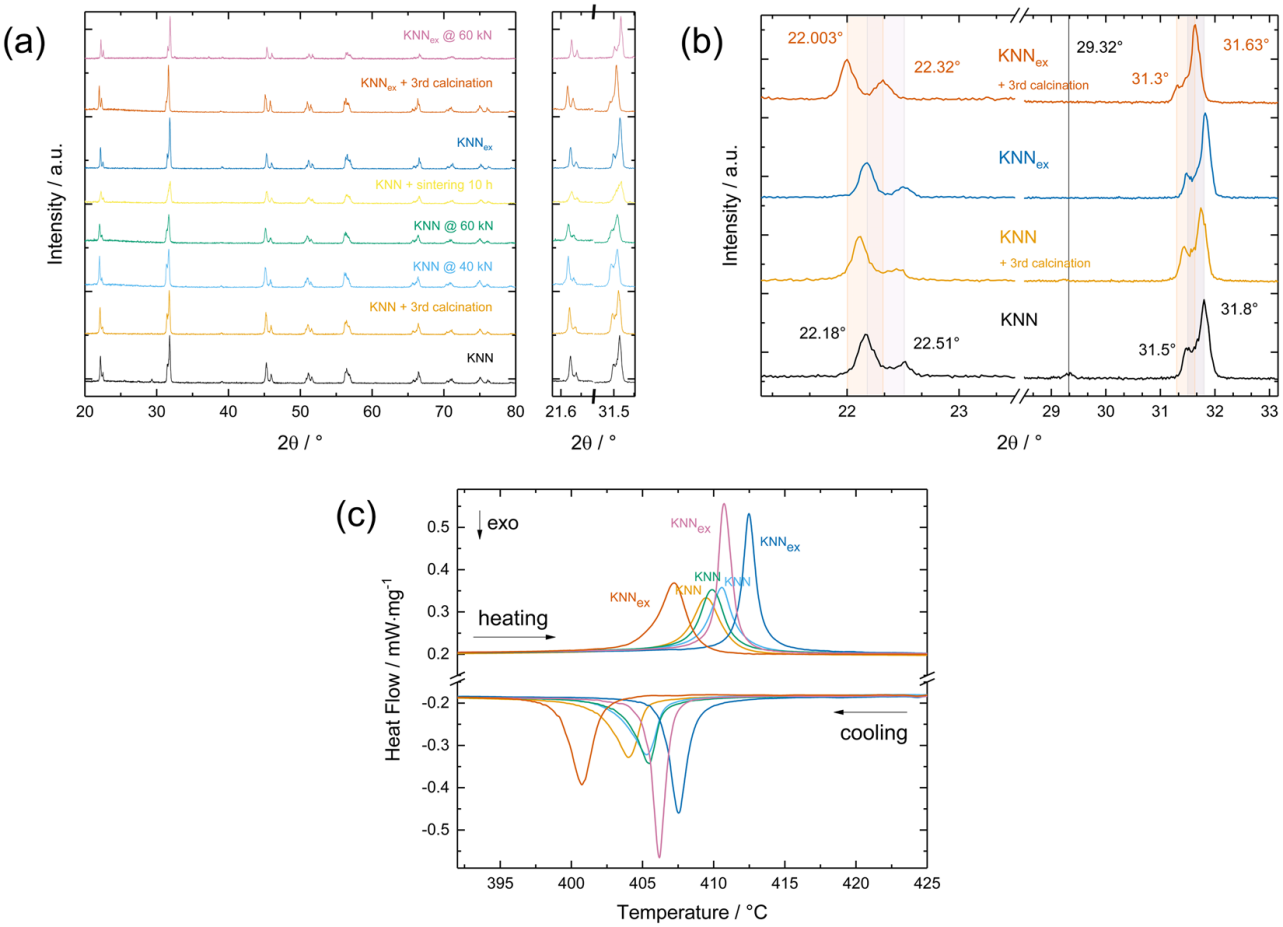
Excess alkali addition: KNN and KNN_ex_ bulk ceramics. Samples are represented by the same colour in all plots. (**a**) X-ray diffractrograms of KNN and KNN_ex_ bulk samples resulting from different processing conditions. Overall comparison of influences and process window. (**b**) Visualisation of process sensitivity for selected samples from (**a**) by analysing the (011)/(100) and (002)/(020)/(111) orientations. A third calcination affects peak positions and orientations. (**c**) Influence on the thermal properties. Differential scanning calorimetry measurements of selected samples from (**a**).

Figure 3(a,b) show XRD spectra (data resolution < 0.008°) of varying samples at room temperature representing different fabrication batches, which were acquired after sintering in air. Apart from differing batches, the presented samples can be distinguished by the number of process steps and/or additional annealing conditions, pressing forces and sintering techniques (differing colours). Samples with undesired secondary phases after processing can clearly be distinguished from those samples, which do not show undesired phases (Fig. 3(a), black line). Although a reproduction of sample quality is difficult as has been mentioned before the calculated positions for potassium sodium niobate peaks (COD, #2300499^60^) for *K*_0.5_*Na*_0.5_*NbO*_3_ at room temperature could be verified for all tested KNN samples and a parameter range can be demonstrated within which potassium sodium niobate can be fabricated reliably without any second or multi niobate phases (Fig. 3(a)). However, the set of process parameters has a major impact on the position of the main reflexes, preferred orientations and the phases which will develop during the sintering process. The process remains highly sensitive to differences at all times and this holds true for the addition of excess alkali metals as well. In particular, this can be seen from Fig. 3(b), as a third calcination causes a strong peak shift and changes the preferred orientations in both cases. As shown, there is especially strong suppression of the (002) direction in favour of the (020) and (111) directions (space group *Amm*2) for KNN_ex_. For KNN the effect is less pronounced and in the same way KNN shows a smaller peak shift of 0.06° compared to 0.18° in KNN_ex_. Consequently, longer annealing times lead to enlarged unit cell sizes at all times, but to different extents for the different compositions. Other possible differences include those parameters which are analysed frequently i.e. annealing time^11,61^, as well as parameters which are discussed seldom e.g. the applied pressing force (cf. supplementary material) and milling time. Moreover, it can be verified from Fig. 3(b) that the addition of excess alkali metals introduces a preferred orientation into the sample even without a third calcination. This preferred orientation for KNN_ex_, which was suggested from the SEM analysis (cf. Fig. 2(h)) was obtained for a comparably low pressing force of 40 kN, which did not result in a preferred orientation in the KNN sample for the very same processing conditions. However, those parameters are not sufficient to control the abnormal grain growth in KNN and also samples do not show improved sinterability with repeated calcination, as has been reported before^35,39^ (cf. supplementary information).

It is well known that *K*_0.5_*Na*_0.5_*NbO*_3 _undergoes three phase transitions which have been identified to occur around ~ −120 °C, 200 °C and 410 °C^62^. Hereby, the exact temperatures depend on the composition of the sample. The first one describes the phase change from a rhombohedral to an orthorhombic (R-O) configuration, the second one is a first-order phase transition from an orthorhombic to a tetragonal configuration (O-T) and the third one describes the temperature-induced change from a tetragonal to a cubic (T-C) lattice. In the following especially the last phase transition will be discussed as it exhibits the largest latent heat, thus it is most interesting with respect to caloric application. Figure 3(c) shows differential scanning calorimetry (DSC) curves of the tetragonal to cubic (T-C) phase transition for representative KNN and KNN_ex_ samples from Fig. 3(a). The thermal analysis shows that an introduction of excess alkali metals increases the latent heat during phase transition significantly and may also induce a shift of the phase transition. Hereby, the transition sharpens and shifts towards higher temperatures for samples with improved material properties and broadens and shifts towards lower temperatures for excess alkali samples which have not been processed along the optimum process parameters. In general, the broadening and the shift are a consequence of the broadened statistical occurrence of phase transition start temperatures, which is caused by varying grain sizes and microstructure inhomogeneity.

### Compatibility and functional fatigue

**Figure 4:**
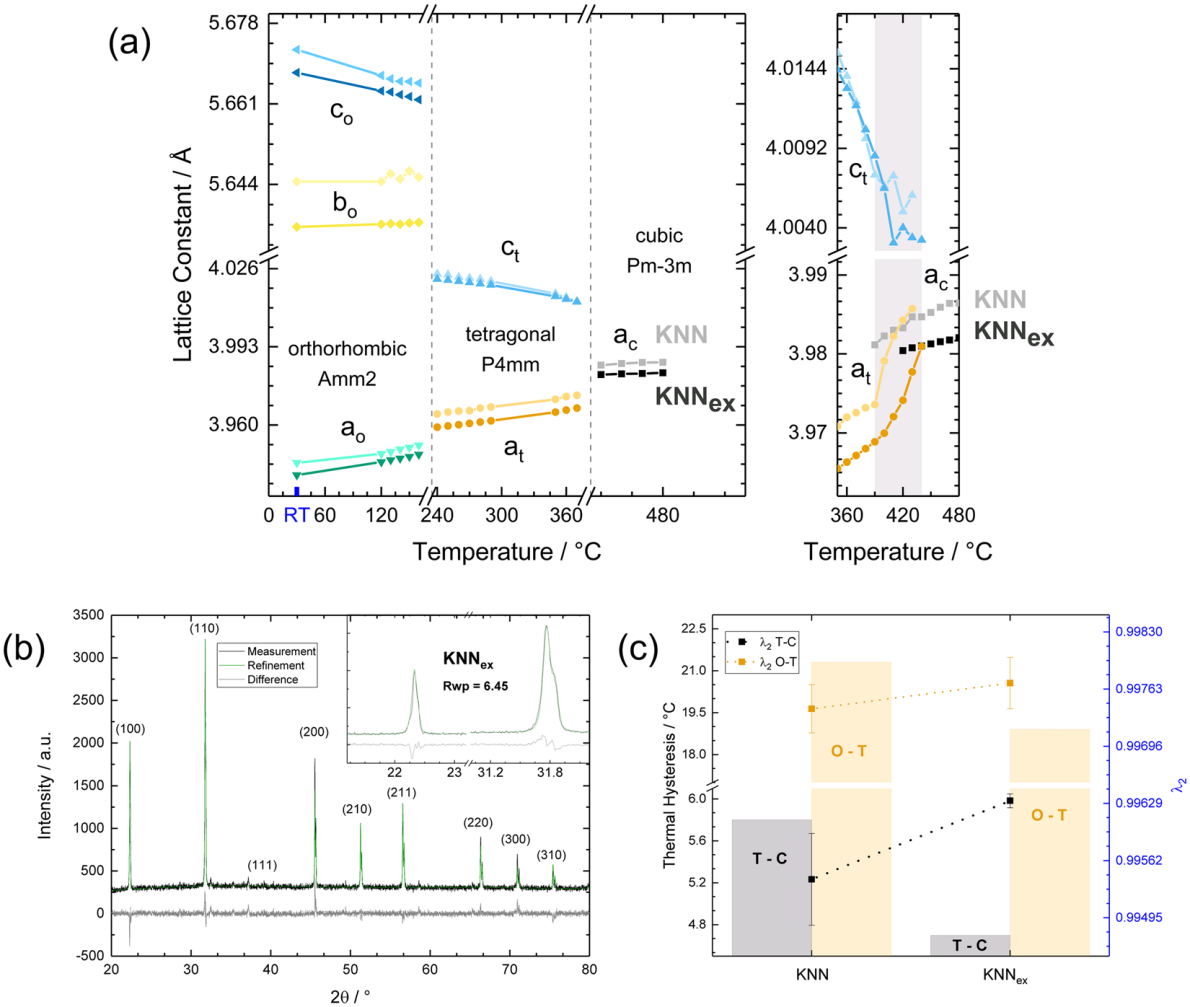
Temperature-dependent lattice constants determination, refinement and compatibility results. (**a**) Left: temperature-dependent development of the lattice constants in KNN and the best performing KNN_ex_ sample respectively. Right: fitting results for the phase transition region for the tetragonal to cubic phase transition. (**b**) Rietveld refinement results for the KNN_ex_ sample shown in (**a**) and enlarged depictions of the fit for the (100) and (110) orientations. (**c**) Thermal hysteresis and phase compatibility (λ_2_) correlation for KNN and KNN_ex_.

Although the phase transitions of KNN are very well known to exist, there is very little verified information on the high temperature cubic phase and the phase transitions of KNN available due to the mentioned obstacles. Often the reported results contradict each other in terms of applied crystallographic symmetries e.g. space group and crystallographic configuration at room temperature^60,61^. Yet, the application of the compatibility theory requires the accurate determination of lattice constants^63,64^. Briefly, the obtained lattice constants are used to calculate the three eigenvalues *λ*_*n*_, *with n* = 1,2,3 of the transformation stretch matrices of participating phases (initial and transformed phases) in a way that *λ*_1_ ≤ *λ*_2_ ≤ *λ*_3_^63^. Hereby, the middle eigenvalue *λ*_2_ is of special importance, as *λ*_2_ = 1 implies that transition layers with no elastic energy exist between those phases i.e. it describes the existence of perfect unstressed interfaces between the participating phases. Moreover, it is stated that the energy in the transition layer grows strongly as the deviation of *λ*_2 _from 1 increases. Therefore, the compliance with this purely geometric condition can decrease functional and structural fatigue and influence hysteresis dramatically^48^ by the elimination of stressed interfaces during a phase transition. In the specific case of a cubic to tetragonal lattice structure the middle eigenvalue *λ*_2_ can be calculated by using the simple relationship *λ*_2_ = *a*_*cubic*_/*c*_*tetra*_ due to the given crystal lattices. Temperature-dependent XRD spectra were repeatedly recorded between room temperature (RT) and 500 °C and Rietveld-fitted resulting in the temperature-dependent lattice constant development which is shown in Fig. 4(a). All the visible peaks, which do not belong to the KNN diffractogram were shown to originate from the heating stage (cf. supplementary material). The goodness of the refinement was judged according to graphical match^65^ and the weighted profile R-factor R_wp_, which is a very direct index of discrepancy that is calculated from the square root of the minimised quantity and scales with the weighted intensities^66^. It can be seen that the microstructural differences between KNN and KNN_ex_ are not the only major differences between those material compositions. The major differences in lattice constants reveal a change on the atomic scale from yet another perspective, which is a remarkable shrinkage in unit cell size for KNN_ex_. This is caused by the partial filling of vacancies which arise from alkali deficiency. Ionic bond lengths are shorter than the atomic radii hence a reduced unit cell volume points to a very favourable (closely bonded) configuration. Remaining vacancies most probably still occur due to alkali or oxygen deficiency, as no niobium deficiency could be found experimentally in any of the fabricated samples (cf. supplementary information). Yet, with respect to the fact that the peak positions and accordingly the unit cell size were confirmed for KNN according to theoretical calculations^60^, filling of vacancies cannot be the only explanation here. It is assumed that more complex reasons e.g. favourable tilt of oxygen octahedrons or favourable arrangement of vacancies might play a role here. Figure 4(b) shows the high temperature phase fit for the shown excess alkali sample from Fig. 3(a). The fit yielded a weighted profile R-factor R_wp_ of *R*_*wp*_ = 6.45, while the atomic positions were fixed. Taking this and the minor difference curve into account the results are in very good agreement with the measurement data (Fig. 4(b) inset). The suggested space groups are *Amm*2 at room temperature, *Pmm*4 in the tetragonal phase and *Pm* − 3*m* for the cubic phase as shown in the corresponding regions of the diagram (Fig. 4(a)). Those space groups are chosen in agreement with theoretical mode-symmetry calculations^60^ and Ahtee & Glazer’s phase diagram^62^. A complementary description of the fitting parameters can be found in the Methods section and a fit result for KNN is provided in the supplementary material. As a result of the good agreement between model and experimental data, the fitting parameters that were obtained for KNN were used as a starting point for all other samples (*R*_*wp*_ = 5.71). The procedure allows for the determination of lattice constants, which can be used for the calculation of the middle eigenvalues *λ*_2_ as mentioned before (Fig. 4(c)). The differences in hysteresis are described correctly by the compatibility theory and it is especially worth mentioning, that the tetragonality increases strongly for the orthorhombic to tetragonal phase transition, while *λ*_2_ improves and the latent heat increases (cf. supplement). However, the *λ*_2_ values are overall very close to 1 and the theory is subject to the restrictions of microstructure at this point.

**Figure 5:**
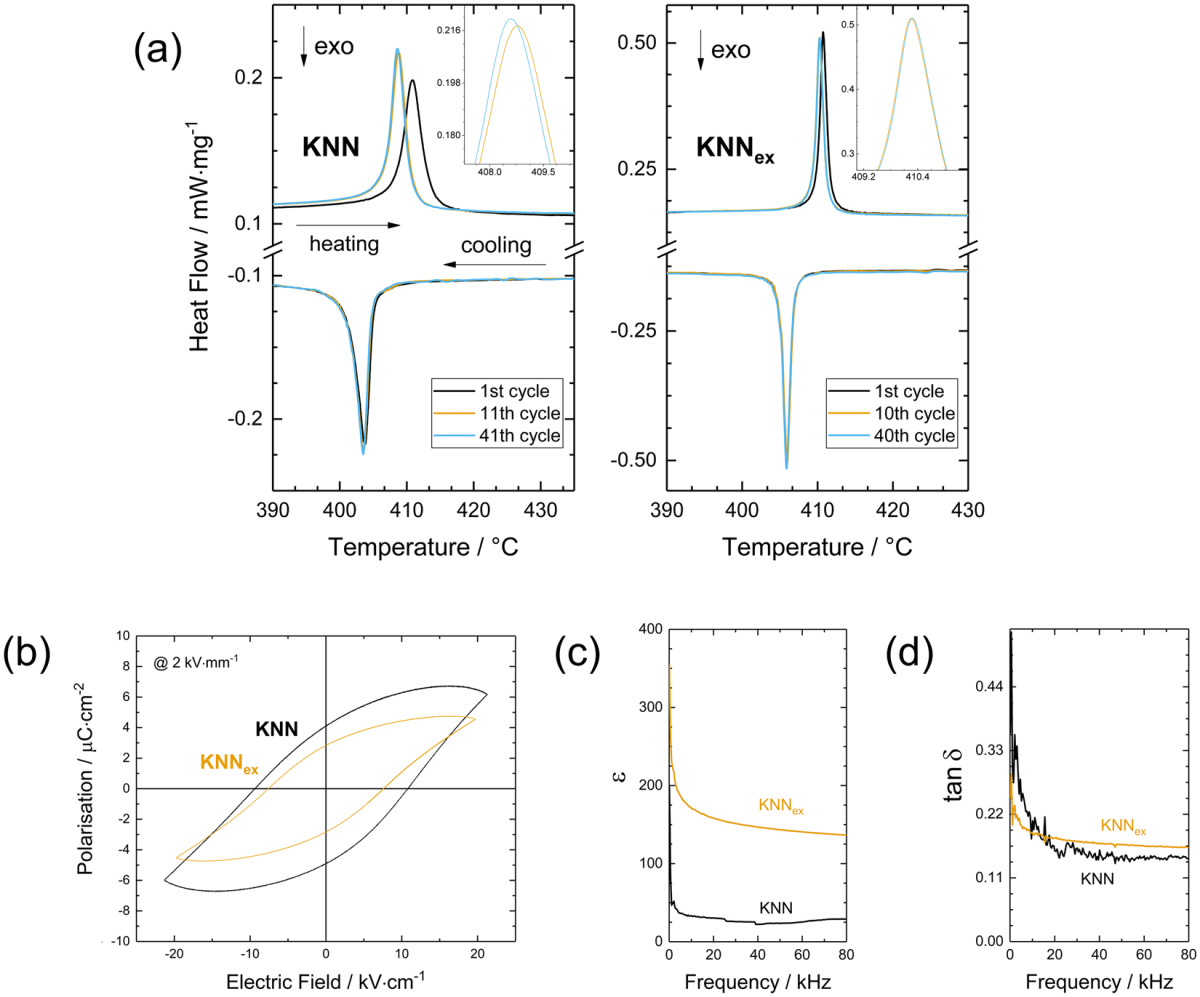
Improvement of the functional properties. (**a**) Thermal cycling of the analysed KNN and KNN_ex_ bulk ceramics with enlarged phase transition peak (inset) reveals enhanced cyclic stability for KNN_ex_ after 40 thermal cycles. The presented cycles were performed at 10 K/min ramps and the cycles in between were performed at accelerated 50 K/min temperature ramps. The electrical characterisation includes the dielectric hysteresis recorded at 2 kV/mm (**b**), the dielectric permittivity ε (**c**) and the dielectric loss factor tan δ (**d**) given for a frequency sweep from 0 up to 80 kHz.

With regard to fatigue behaviour, the predicted improvement is already clearly visible after a few thermal cycles (Fig. 5(a)). Thermal cycling was executed with the help of a differential scanning calorimeter. The shown cycles were measured at 10 *K*/*min* ramps to enable detailed analysis, while the cycles in between were driven at accelerated degeneration conditions (50 *K*/*min* ramps). The increase in bulk energy, the associated strain and higher probability for irreversible deformation for the lower *λ*_2_ value in KNN are recognisable as pronounced difference in hysteresis. This can be assumed to originate from the fact that microstructural effects superimpose all other effects here, for example the geometric match of participating phases. In specific, smaller grains result in a higher number of transition layers, which will therefore accumulate in occurrence in those regions independently from match or mismatch. In the simplest case, this means an increased probability for misfit and defect formation. And this is yet not the only disadvantage from grain size inhomogeneity. Apart from differing transition temperatures, there is increased probability for misfit at interconnections between those small and large grains and of course more grain boundary diffusion. For KNN_ex_, which is a better resemblance of the predictions of the compatibility theory as for the homogeneous microstructure, the sample with a *λ*_2_ value closer to 1 shows no visible fatigue after thermal cycling. In contrast, the KNN sample exhibits visible fatigue after only 40 cycles. These combined grain size and compatibility effects are mirrored by the electronic performance of the material as well (Fig. 5(b,c)). Smaller grain sizes and especially microstructural heterogeneity show deteriorated dielectric response, which is in agreement with other studies^67^. Bigger, homogeneously distributed grain sizes on the other hand cause improvements in electrical behaviour e.g. a narrowed hysteresis for KNN_ex_ which corresponds to a decreased amount of required input work in application e.g. the electrocaloric effect. Furthermore, KNN_ex_ exhibits improved saturation properties and could be verified despite a very low density. It is assumed that microstructural inhomogeneity impedes an easy alignment of the polarisation vectors due to misfit at the grain boundaries, which causes electrical losses especially at low frequencies. If a sample actually fulfils the compatibility conditions in terms of microstructure homogeneity, major improvements can be achieved. Although the value for the dielectric permittivity ε is low, powder morphology control alone was able to yield an increase by a factor of 4 without the need of complex doping. Hence, it can be concluded that the improvement of the functional properties originates from a grain size effect: larger grains and a more homogeneous microstructure cause this significant change in the material properties, rather than processing technique and density of the material do. Consistently, the persisting low density (poor sinterability) is primarily monitored through the dielectric loss tan δ (Fig. 5(d)), which is the only material characteristic that does not improve in these experiments by any of the applied means. Secondarily, the low density affects the permittivity *ε* and the spontaneous polarisation *P*_*S*_ as well, which can be recognised by the low values for *ε* and *P*_*S*_ even in the case of KNN_ex_. A low density leads to a small overall dipole moment, as less dipoles are available, while the leakage current increases. It is noted that experiments on the use of sintering aids have shown to be promising e.g. the addition of *CuO*^39^ and *Fe*_2_*O*_3_^68,69^.

## Conclusion

In this work abnormal grain growth was identified to originate from the precursor powders and it was shown that even under simple conditions, abnormal grain growth can be suppressed. Most importantly, the relation between milling duration and calcination duration needs to be very high i.e. the milling duration is required to be very long in contrast to the calcination duration. Secondly, the procedure needs to be repeated for that the process of abnormal grain growth is self-accelerating. The more abnormal grains occur, the more will develop consecutively as for the change in the edge free energy. Hence, the precursor powder needs to be milled for long times of at least 24 h at an adequate rotation speed to ensure homogenisation of grain sizes after every calcination to level the temperature-triggered inequalities between the A-site elements potassium and sodium. On the basis of the DLS and SEM analysis it could be demonstrated that this successful suppression of abnormal grain growth is the reason for a dramatic improvement of material performance in terms of microstructure homogeneity and that it can be achieved by controlling the powder morphology solely. It is noted that the achieved improvement is remarkably high in regard to the fact that it relies on process control only without adding complex alloys to the synthesised KNN. Furthermore, predictions on sample quality can be made from the analysis of the powder morphology, as the properties of the KNN grains in the powder are mirrored by all subsequent steps. Homogeneous grain size distributions in the powder directly result in homogeneous grain size distributions in the sintered bulk material and the material properties on the nanoscale can hardly be influenced efficiently in the retrospective. This grain size effect originates from the calcined powder, not from the sintering process, which is more likely to influence density and overall grain size growth. Additionally, the suppression of abnormal grain growth causes a significant improvement in most of the functional properties with respect to electronic and caloric applications. Here, the positive effects of powder morphology control range from an increased dielectric permittivity and narrowed hysteresis to the minimisation of fatigue and increase in latent heat. However, all of this is achieved more or less independently from the density of the material, which remains to be improved.

## Methods

All of the presented samples were produced according to the conventional solid state route. The used precursor powders *Na*_2_*CO*_3_(ROTIMETIC 5 N, anhydrous, Carl Roth), *K*_2_*CO*_3_ (ROTIMETIC 4 N5, Carl Roth), *Nb*_2_*O*_5_ (4N, Sigma-Aldrich) were obtained from Carl Roth and Sigma-Aldrich. All the powders were dried at 200 °C for 10 minutes prior to usage and ball milled in alumina crucibles filled with hexane (130–150 rpm, min. 24 h). Subsequently the powders were calcined (850 °C–900 °C) for 6 h and the procedure was repeated for both steps. After a third milling step the calcined powders were mixed with 3 wt.-% of polyvinyl alcohol (PVA) and left to dry in a drying furnace (80 °C, 24 h). Afterwards the powders were sieved with a 125 μm sieve and pressed into green bodies (disks) with a diameter of d = 10 *mm* and varying thicknesses. The sintering procedure varied in duration and temperature (1060 °C–1130 °C, 6 h–24 h) and yielded sintered ceramic pellets. To determine the structural properties XRD measurements were executed using a Rigaku SmartLab 9 kV X-ray diffractometer with CuK_α_ radiation $(\lambda =1.5406\,{\rm{\AA }})$ and obtained diffractograms were fitted with the TOPAS-Academic V6 program by Coelho Software using the Rietveld refinement method^70^. To describe the peak shape a symmetric Thompson-Cox-Hastings pseudo-Voigt (TCHZ) peak profile function was used and selected parameters i.e. background level, scale factor, specimen displacement, lattice parameters and preferred orientation (optional) were refined iteratively. A correction for peak asymmetry was applied according to Finger *et al*. and co-workers^71^ to account for axial divergence. Also, the theoretical density was obtained by Rietveld refinement. For the examination of the microstructure a Carl Zeiss SUPRA 55VP scanning electron microscope was used to analyse the samples. Thermal analysis and cycling were executed with a NETZSCH DSC 204 F1 Phoenix differential scanning calorimeter and the NETZSCH software Proteus 7.1.0. With respect to analysis the thermal hysteresis Δ*T* was calculated using the equation ${\rm{\Delta }}T=1/2({T}_{s,i}+{T}_{f,i})-({T}_{s,t}+{T}_{f,t}))$, wherein start (indexed with s) and finish (indexed with f) temperatures of the participating phases T_s,i_, T_f,i_ (initial phase), T_s,t_ and T_f,t_ (transformed phase) were obtained by the application of the tangent method. The electrical characterisation was performed with the help of an Agilent Technologies 4294 A impedance analyzer for the measurement of ε and tan δ and an aixACCT aixDBLI double-beam laser interferometer system was utilised to record P-E hysteresis loops. The determination of powder particle sizes was performed in ethanol using a Beckman Coulter DelsaNano C particle size analyzer. The density of the samples was determined through the correlation *ρ* = *m*/*V*, where ρ is the density, m is the mass of the sample and V the volume of the sample.

## Supplementary information

supplementary information

## Data Availability

The data that supports the findings of this study is available from the corresponding author on reasonable request.
