# Supplementary material for: Suppression of abnormal grain growth in K0.5Na0.5NbO3: phase transitions and compatibility
Source: Sci Rep. 2019 Dec 24;9:19775. doi: 10.1038/s41598-019-56389-9 (PMC6930306; doi:10.1038/s41598-019-56389-9)
Supplement: Supplementary file 1 — supplementary information [file 41598_2019_56389_MOESM1_ESM.pdf]

Suppression of abnormal grain growth in $K_{0.5}Na_{0.5}NbO_3$: phase transitions and compatibility

Patricia Pop-Ghe[1,*], Norbert Stock[2], Eckhard Quandt[1]

[1] Inorganic Functional Materials, Kiel University, 24143 Kiel, Germany

[2] Inorganic Chemistry, Kiel University, 24143 Kiel, Germany

* Corresponding author: ppg@tf.uni-kiel.de

Supplementary material

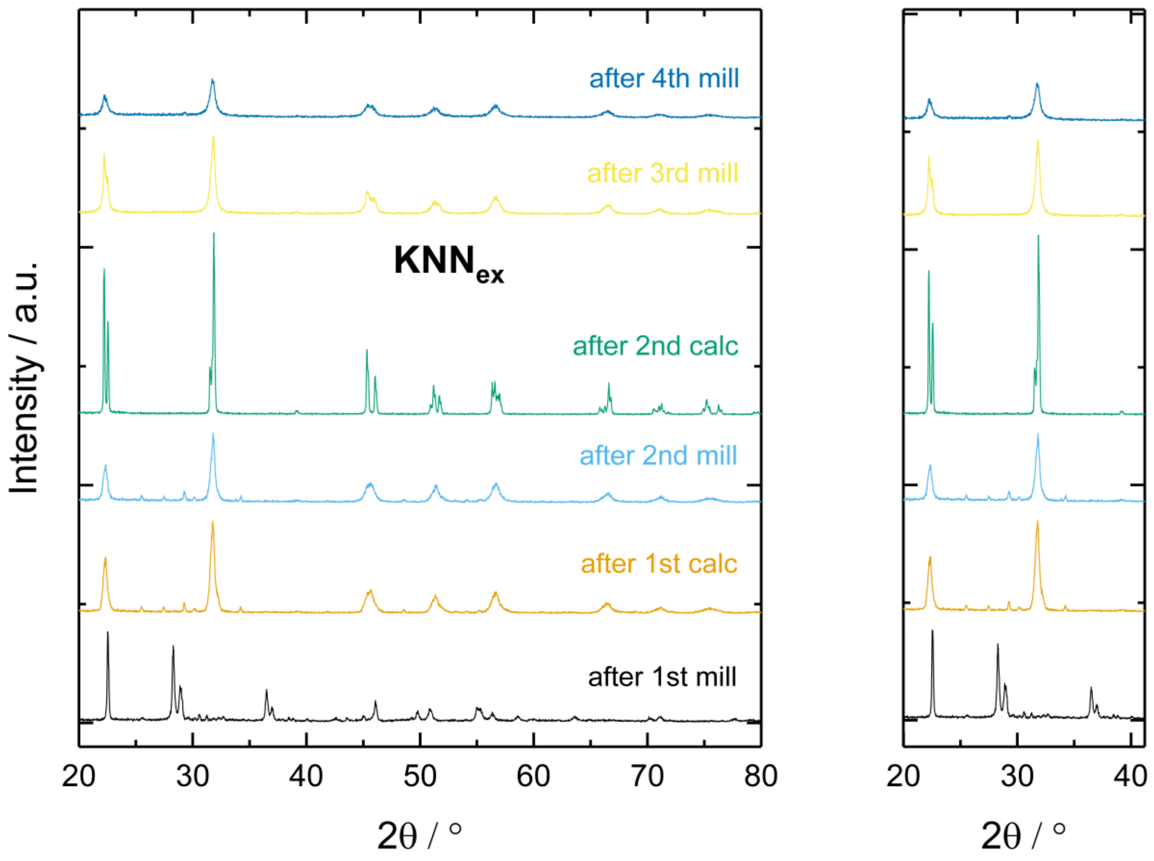

Supplementary Figure S1. Additonal repetitions of calcination and milling cause a strong detereoration in peak sharpness in the recorded diffractogram (the presented fourth milling includes a prior calcination). This can be seen from the decreased intensities of the peaks, as well as the discriminability of different orientations. The enlargement given on the right shows that the main reflexes are already indistinguishable for four repetitions, while sintering after the second milling leads to strong inhomogeneities in the bulk ceramic.

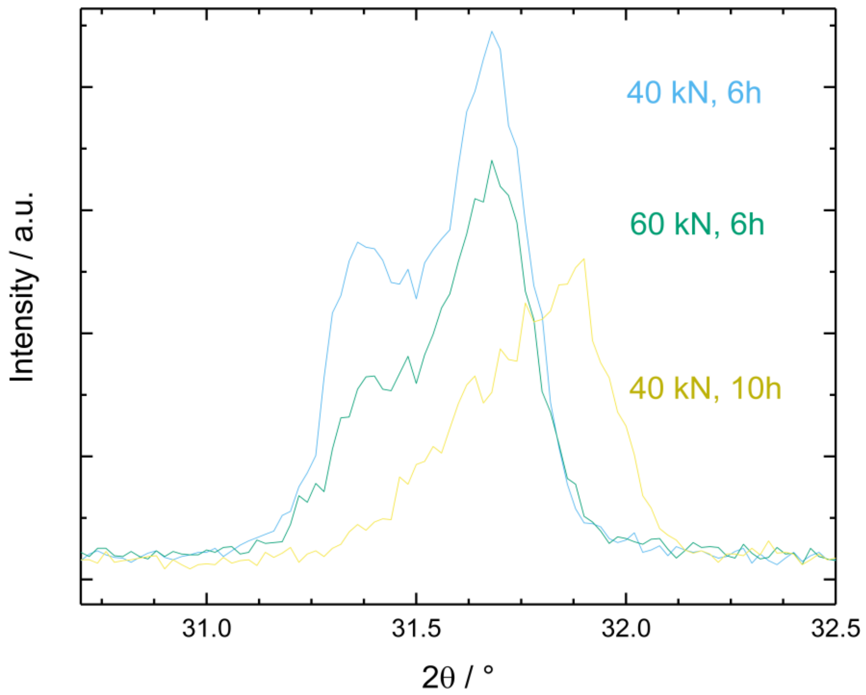

Supplementary Figure S2. The applied pressing force varies strongly, mostly without any discussion of the influence on the sample properties. A representative comparison of pure KNN samples reveals that higher pressing forces (green line) may suppress orientations in the sample and may thus introduce preferred orientations compared to lower pressing forces (light blue line). The same pressing force might result in altered material properties in differently sintered samples and it has to be taken into account that results may vary strongly on the basis of the applied force. Colours correspond to the plot in the main publication (cf. Fig. 3).

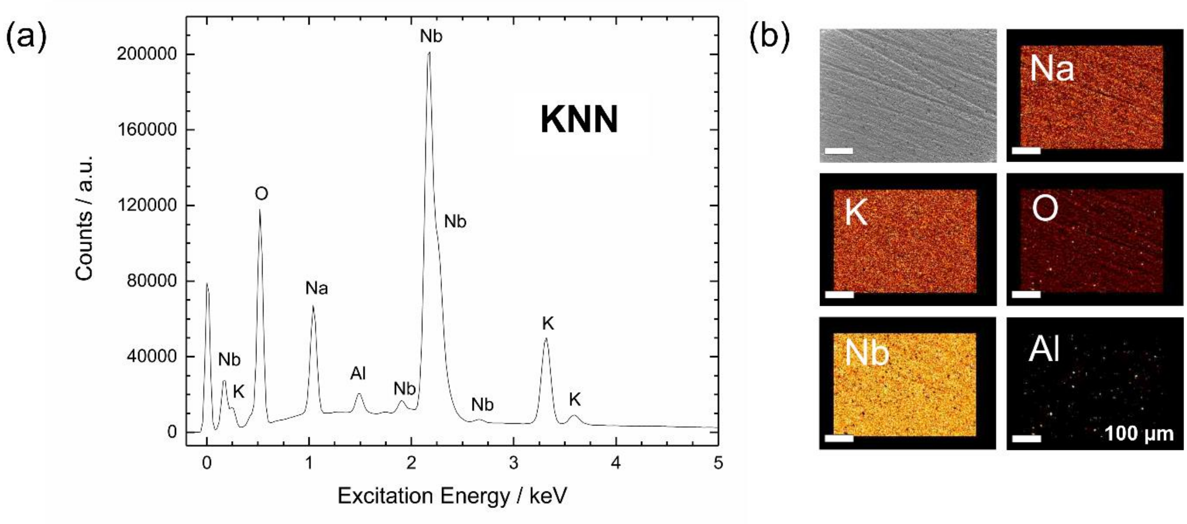

Supplementary Figure S3. Representative energy-dispersive spectroscopy (EDS) measurement and mapping of a KNN sample. (a) Despite the fact that the XRD spectra did not show any other phases an EDS shows the contamination of the samples with aluminium oxide particles (Al_2O_3), which is caused by the milling in alumina crucibles with alumina grinding balls. Although the samples were tested for elemental aluminium, the combined occurrence of aluminium and oxide in the representative EDS mapping allows for the conclusion that the sample is contaminated with a statistical amount of alumina, whose extent is dependent on the milling time of the powder samples. The fabricated samples were milled for comparable amounts of time and at comparable speed, therefore the amount of alumina in the samples is assumed to be more or less constant except for statistical deviation. (b) Interestingly, the EDS mapping unveils that the surface polishing has a stronger influence on the sodium and oxygen atoms than it does on potassium and niobium. The polishing trenches are clearly reproduced in the sodium

and the oxygen mapping, but faint for the other tested elements. These findings are in favour of the assumption of chemical heterogeneity, but need more analysis for validation.

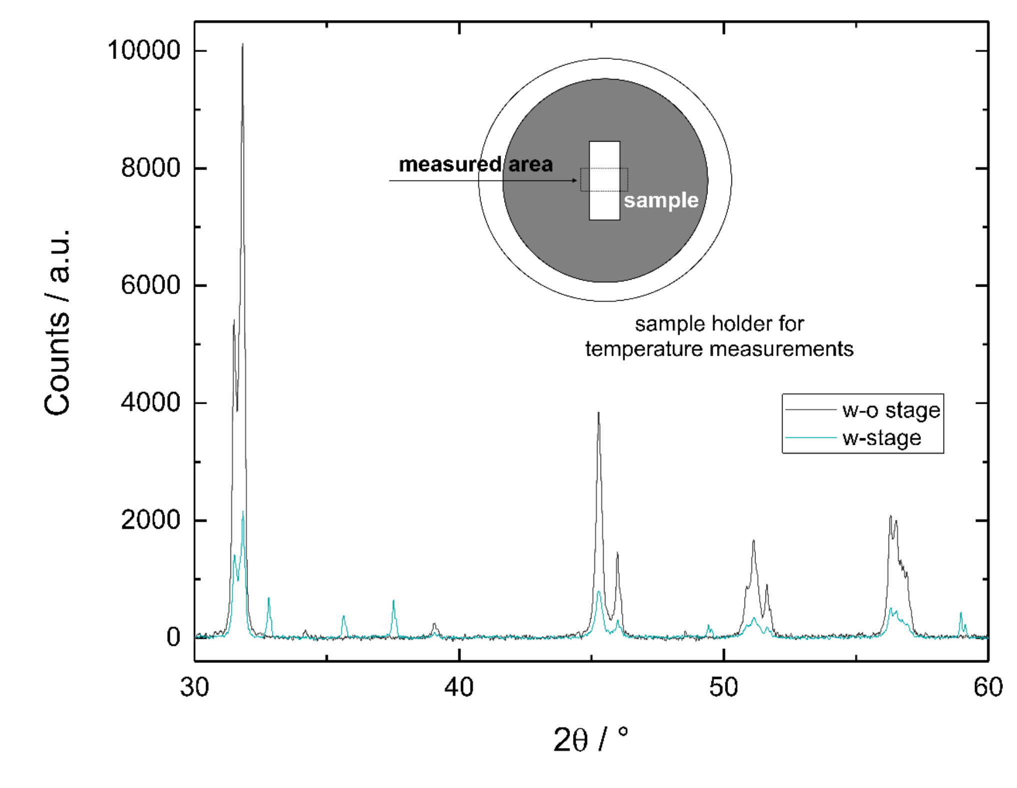

Supplementary Figure S4. The occurrence of unindexed peaks in the presented fit can be explained by the small dimensions of the measured sample combined with the heating stage needed for temperature-dependent measurements. The comparison between X-ray difractograms with (w-stage) and without (w-o stage) heating stage at room temperature shows, that the ceramic heating stage itself shows crystallinity.

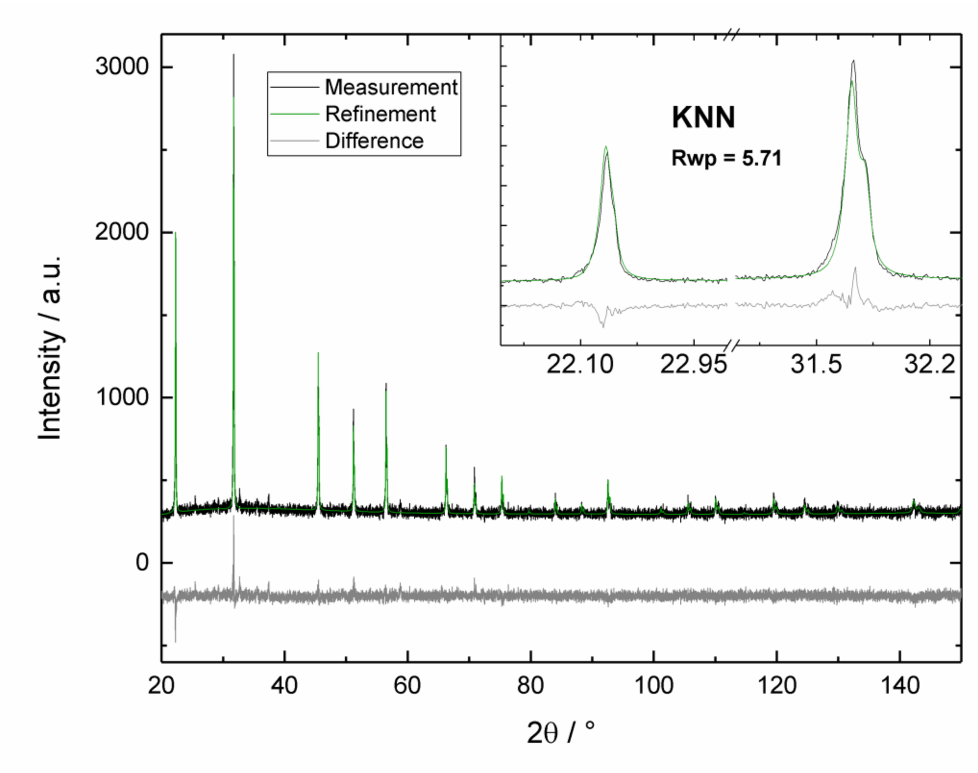

Supplementary Figure S5. Rietveld refinement results for the presented KNN sample and enlarged depictions of the fit for the (100) and (110) orientations in the inset. The measurement data is given in black, the refinement is shown in green and the difference between the two is given by the grey line (difference curve). Relative intensities are given correctly.

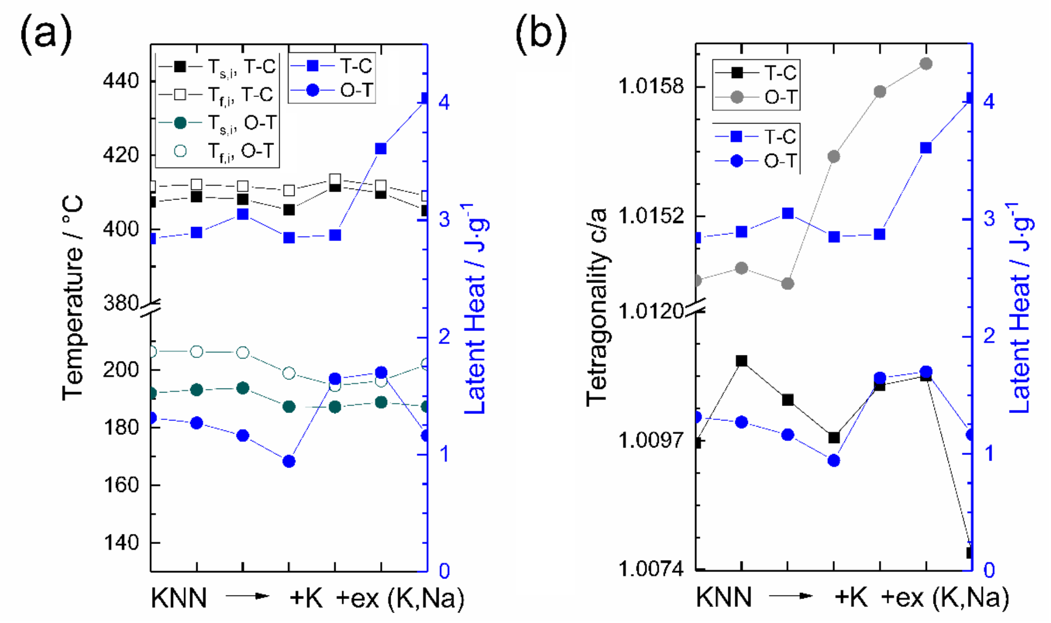

Supplementary Figure S6. Correlation of phase transition temperatures, tetragonality and latent heat. (a) Start (indexed with s) and finish (indexed with f) temperatures of the participating phases (initial phase indexed with I, transformed phase indexed with t) in transition vs. composition and latent heat given for the tetragonal to cubic (T-C) and orthorhombic to tetragonal (O-T) transitions. The addition of the chosen amount of excess alkali metals leads a narrowing of the hysteresis, as the offset between start and finish temperature narrows. At the same time, higher latent heats can be achieved. For the addition of 5 mole-% potassium excess only (+K), deteriorated material properties are demonstrated i.e. a lower latent heat than in KNN correlated to a smaller tetragonality and a broad thermal hysteresis. (b) Tetragonality vs. composition and latent heat given for T-C and O-T transitions. The values for the tetragonality are given for the edge of the phase transition for differently processed KNN and KNN_{ex} samples. As can be seen from the graph, for KNN_{ex} the tetragonality of the orthorhombic to tetragonal transition increases strongly, as does the latent heat. This development stops, when a third calcination is applied (last data point).

**Processing route**	**Density after sintering [g/cm³]**
Two calcinations and three millings<br><br>Precursor drying at 80°C for 24 h<br>Initial milling at 150 rpm for 24 h<br>Calcination at 900°C for 6 h<br>Repetition of the two prior steps<br>Milling at 150 rpm for 24 h<br>Addition of 3 wt.-% polyvinyl alcohol<br>Drying for 24 h<br>Sieving - 125 µm sieve<br>Pressing for 210 s at 60 kN<br>Sintering at 1080°C for 6 h	3.334<br>equals 74 % of the theoretical density of 4.5188 g/cm³
Repeated calcination<br>*(three calcinations and three millings)*<br><br>Precursor drying at 80°C for 24 h<br>Initial milling at 150 rpm for 24 h<br>Calcination at 900°C for 6 h<br>Repetition of the two prior steps	2.908<br>equals 64 % of the theoretical density

Milling at 150 rpm for 24 h<br>+ *Calcination at 900°C for 6 h*<br>Addition of 3 wt.-% polyvinyl alcohol<br>Drying for 24 h<br>Sieving - 125 µm sieve<br>Pressing for 210 s at 60 kN<br>Sintering at 1080°C for 6 h	

Supplementary Table S7. Exemplary process parameters and density after sintering of presented samples. The determination of the density is described in the Methods section. The theoretical density was determined by Rietveld refinement.
